# Draft Genome Sequence of an Atypical Strain of *Streptococcus pneumoniae* Serotype 19A Isolated from Cerebrospinal Fluid

**DOI:** 10.1128/genomeA.00277-16

**Published:** 2016-04-21

**Authors:** Antonio Ali Perez-Maya, Rosa Maria Hinojosa-Robles, Jose Ramon Barcenas-Walls, Augusto Rojas-Martinez, Hugo Alberto Barrera-Saldaña, Rocio Ortiz-Lopez

**Affiliations:** a Universidad Autónoma de Nuevo León, Facultad de Medicina, Departamento de Bioquímica y Medicina Molecular, Monterrey, Nuevo León, Mexico; b Universidad Autónoma de Nuevo León, Hospital Universitario José Eleuterio Gonzalez, Monterrey, Nuevo León, Mexico; c Universidad Autónoma de Nuevo León, Centro de Investigación y Desarrollo en Ciencias de la Salud, Unidad de Genómica, Monterrey, Nuevo León, Mexico

## Abstract

We present here the draft genome sequence of *Streptococcus pneumoniae* strain MTY32702340SN814 isolated in Monterrey, Mexico, from a girl with bacterial meningitis. The strain belongs to the atypical and multidrug-resistant serogroup 19A. This is the first report in the literature of sequence type 3936 (ST3936) in *S. pneumoniae* serotype 19A.

## GENOME ANNOUNCEMENT

*Streptococcus pneumoniae* is associated with significant morbidity and mortality, mainly in children and the elderly (1). The introduction of the 7-valent pneumococcal conjugate vaccine (PCV7) in the United States and other Western countries has significantly reduced the incidence of invasive pneumococcal disease (IPD) by the seven vaccine strains (2, 3). However, the increasing global emergence and rapid spread of serotypes not included in PCV7, as well as the rise in the number of cases of invasive infections caused by these serotypes, are a serious concern (3, 4). Particularly, serotype 19A sequence type 320 (ST320) is currently considered an emerging strain associated with IPD and multidrug resistance (MDR) (5–8).

*S. pneumoniae* MTY32702340SN814 (ST3936, serotype 19A) is a multidrug-resistant strain isolated from a cerebrospinal fluid sample from a 12-year-old girl in 2014 at the Hospital Regional Materno Infantil in Northeast Mexico. This patient suffered acute meningitis and died on the day of admission. Strain MTY32702340SN814 was resistant to penicillin, trimethoprim-sulfamethoxazole, and tetracycline (9).

Additionally, the allelic profile (ST3936) of our isolate has been compared with those in the reference multilocus sequence typing (MLST) database (http://spneumoniae.mlst.net/), and only three pneumococcal isolates had a profile identical to that of our strain. The above-mentioned three strains (from serotype 19A-ST3936) were from U.S. patients who had developed pneumonia. Furthermore, we searched for isolates in our region that had allelic profiles similar to ours, with at least 6/7 matches, and we found single-locus variants from ST230, which differs in *xpt* (xanthine phosphoribosyltransferase), and ST4511, which differs in *ddl* (d-alanine–d-alanine ligase), both recovered from patients with invasive disease in the United States. This occurrence may represent a new clonal group and therefore, it will be necessary to continue molecular surveillance to monitor the ability of serotype 19A-ST3936 to spread within the region.

The genome was sequenced using the Illumina MiSeq platform (250-bp paired-end reads). A total of 2,147,688 reads were obtained, providing about 57-fold genome coverage. *De novo* assemblies were performed using the SPAdes genome assembler version 3.5.0 software (10). The unclosed draft genome of MTY32702340SN814 was assembled into 236 contigs (≥500 bp), with a total length of 2,191,102 bp and a G+C content of 40.9%.

The genome assembly was annotated using the NCBI Prokaryotic Genome Annotation Pipeline (11). Additionally, a total of 2,403 genes were predicted, which included 2,073 coding sequences (CDSs), 5 rRNAs (three copies of 5S, one copy of 16S, and one copy of 23S rRNA genes), 48 tRNAs, and 3 noncoding RNAs (ncRNAs).

### Nucleotide sequence accession numbers.

This whole-genome shotgun project has been deposited at DDBJ/ENA/GenBank under the accession no. LSSU00000000. The version described in this paper is version LSSU01000000.
